# What is the impact of local control in Ewing sarcoma: analysis of the first Brazilian collaborative study group – EWING1

**DOI:** 10.1186/s12885-017-3391-5

**Published:** 2017-06-15

**Authors:** Ricardo G. Becker, Lauro J. Gregianin, Carlos R. Galia, Reynaldo Jesus-Garcia Filho, Eduardo A. Toller, Gerardo Badell, Suely A. Nakagawa, Alexandre David, André M. Baptista, Eduardo S. Yonamime, Osvaldo A. Serafini, Valter Penna, Julie Francine C. Santos, Algemir L. Brunetto, On behalf of the Brazilian Collaborative Study Group of Ewing Family of Tumors – EWING1 and the Brazilian Society of Pediatric Oncology – SOBOPE

**Affiliations:** 1 Service of Orthopedics and Traumatology, Hospital de Clínicas de Porto Alegre (HCPA), Rua Ramiro Barcelos, 2350, Bairro Santa Cecilia, Porto Alegre, RS 90035-903 Brazil; 2 Department of Pediatrics, HCPA, Universidade Federal do Rio Grande do Sul (UFRGS), Porto Alegre, Brazil; 3 Department of Pediatrics, Hospital São Lucas, Pontifícia Universidade Católica do Rio Grande do Sul (PUCRS), Porto Alegre, RS Brazil; 4 Service of Orthopedics and Traumatology, HCPA, Porto Alegre, RS Brazil; 5 Support Group for Children and Adolescents with Cancer (GRAACC), Universidade Federal de São Paulo (UNIFESP), São Paulo, SP Brazil; 6 Fundação Pio XII, Hospital de Câncer Infantojuvenil, Barretos, SP Brazil; 7 Centro Hospitalario Pereira Rossell, Montevideo, Uruguay; 8 Orthopedics Service, Hospital A.C. Camargo Cancer Center, São Paulo, SP Brazil; 9 Service of Orthopedics and Traumatology, Santa Casa de Misericórdia de Porto Alegre, Porto Alegre, RS Brazil; 10 Orthopedic Trauma Institute, Hospital das Clínicas de São Paulo, School of Medicine, Universidade de São Paulo (USP), São Paulo, SP Brazil; 11 Department of Orthopedics and Traumatology, Santa Casa de Misericórdia de São Paulo (HSCSP), São Paulo, SP Brazil; 12 Service of Orthopedics and Traumatology, Hospital São Lucas, Pontifícia Universidade Católica do Rio Grande do Sul (PUCRS), Porto Alegre, RS Brazil; 13 Hospital das Clínicas de Botucatu, School of Medicine, Universidade Estadual Paulista (UNESP), Botucatu, SP Brazil; 14 Instituto do Câncer Infantil, Porto Alegre, RS Brazil; 15 Instituto do Câncer Infantil, Porto Alegre, RS Brazil

**Keywords:** Ewing sarcoma, Local control, Radiation oncology, Surgery, Bone tumors, Orthopedics

## Abstract

**Background:**

Relapse in localized Ewing sarcoma patients has been a matter of concern regarding poor prognosis. Therefore, we investigated the impact of local control modality (surgery, surgery plus radiotherapy, and radiotherapy) on clinical outcomes such as survival and recurrence in patients with non-metastatic Ewing sarcoma treated on the first Brazilian Collaborative Group Trial of the Ewing Family of Tumors (EWING1).

**Methods:**

Seventy-three patients with localized Ewing sarcoma of bone aged < 30 years were included. The treating physicians defined the modality of local control based on the recommendations of the coordinating center and the patient and tumor characteristics. Possible associations of local control modality with local failure (LF), disease-free survival (DFS), event-free survival (EFS), overall survival (OS), and clinical characteristics were analyzed.

**Results:**

Mean patient age was 12.8 years (range, 2 to 25 years) and median follow-up time was 4.5 years (range, 2.3 to 6.7 years). Forty-seven patients underwent surgery, 13 received radiotherapy, and 13 received both. The 5-year EFS, OS, and DFS for all patients was 62.1%, 63.3%, and 73.1%, respectively. The 5-year cumulative incidence (CI) of LF was 7.6% for surgery, 11.1% for radiotherapy, and 0% for postoperative radiotherapy (PORT) (*p* = 0.61). The 5-year EFS was 71.7% for surgery, 30.8% for radiotherapy, and 64.1% for PORT (*p* = 0.009).

**Conclusions:**

There was a significant effect of local control modality on EFS and OS in the study. Surgery and PORT modalities yielded very close results. The group treated with radiotherapy alone had considerably worse outcomes. This may be confounded by greater risk factors in these patients. There was no significant effect of local control modality on the CI of LF and DFS.

## Background

Ewing sarcoma (ES) is a small round cell malignancy of bone and soft tissue that usually occurs in individuals aged 5 to 20 years. Five-year overall survival (OS) for patients with localized disease ranges from 65 to 75%, while disease relapse after local control reduces survival to less than 25% [1–8]. Multicenter trials have demonstrated the importance of aggressive chemotherapy treatment and local control of the primary tumor. Successful local control rates have improved to 74–93% with the introduction of a multidisciplinary and collaborative approach [9–12].

Current ES treatment includes induction chemotherapy, local control of the primary tumor, and consolidation chemotherapy. Surgery alone or in combination with radiation has traditionally been considered a good choice for resectable ES, while most unresectable tumors have been treated with radiation alone. However, recent studies have reported worse local recurrence and survival rates in patients treated with radiotherapy alone compared to surgery and postoperative radiotherapy (PORT). These findings have been associated with risk factors that are present in irradiated patients [12–19].

For the first time in Brazil, data on local control of ES were analyzed within a single multicenter protocol. We used a cohort of patients with localized ES treated on the EWING1 trial (first Brazilian Collaborative Group Trial for treatment of Ewing sarcoma family of tumors [ESFT]) [20] to evaluate different local control strategies and their association with risk factors, relapse, and survival.

## Methods

### Patient enrollment

The study was approved by the institutional review board of Hospital de Clínicas de Porto Alegre through the Office of Research and Graduate Studies (IRB No. 00000921). All patients signed an informed consent form prior to their inclusion in the EWING1 trial from 2003 to 2010 (original trial, IRB No. 03363, date: October 15, 2003).

Patients with localized ES of bone treated between 2003 and 2010 according to the EWING1 trial were eligible for the study. Patients were allocated to low-risk (LRG) or high-risk (HRG) groups, where high-risk patients were defined as those with unresectable tumors, tumors of the pelvis, and lactate dehydrogenase (LDH) levels ≥ 1.5 times the upper limit of normal (x ULN). Tumor size was assessed on magnetic resonance imaging (MRI) and computed tomography (CT) scans before starting induction chemotherapy and categorized into ≤ 8 cm (small tumors) and > 8 cm (large tumors). Chemotherapy response was defined as good or poor according to the necrosis index (> 95% or ≤ 95%, respectively) [21, 22].

Patients were treated at 15 centers located in 6 states in Brazil, and one in Uruguay. Each center’s institutional review board approved the treatment protocols, and written informed consent was obtained for all patients at enrollment.

### Treatment

In the EWING1 trial, the induction chemotherapy consisted of two courses of ifosfamide/carboplatin/etoposide (ICE) and two courses of vincristine/doxorubicin/cyclophosphamide (VDC), followed by local control. After local treatment, LRG patients received 10 additional alternating cycles of ifosfamide/etoposide (IE) with VDC, while HRG patients received two additional cycles of ICE at the end of the consolidation therapy. Details of the treatment plan and timing of local control have been published previously [20].

Local control modality was defined based on the experience of treating physicians within each participating institution; however, the coordinating center established some criteria based on the patient and tumor characteristics to standardize the choice of local control. Patients with tumors that were amenable to resection with adequate margins, regardless of size, response to chemotherapy, or location, should be treated surgically. Cases with positive surgical margins, in which wide resection was not possible due to high morbidity, should receive PORT. The dose of PORT was defined as 45 Gy for marginal resections and 55.8 Gy for intralesional resections. The presence of necrotic tissue, even in the absence of viable ES cells, was considered incomplete resection and treated with 55.8 Gy. Patients with tumors of the ribs, with a pleural effusion contiguous to a primary lesion, should also receive PORT.

Definitive radiation was given to patients when wide resection could cause high morbidity or mutilation, and in unresectable tumors. Radiation was planned according to the X-ray, CT, and MRI when available. Radiotherapy was delivered to the original tumor volume with a 2-cm margin and a total dose of 55.8 Gy at 1.8 Gy/fraction started during week 11. At the end of treatment, it was established that patients would be followed up every 3 months during the first 2 years, then every 6 months for 5 years, and annually thereafter.

Recurrence was classified as local or systemic. For analysis purposes, any local recurrence was defined as local failure (LF) and systemic recurrence as distant failure (DF). Combined recurrences were included in the systemic group. The classification of the local control modality received by each patient was determined according to all interventions performed at the local tumor site up to and including the start of consolidation therapy. Local control was classified into one of three procedures: surgery, radiotherapy, or surgery plus radiotherapy. Overall survival (OS), event-free survival (EFS), and disease-free survival (DFS) were defined as the time from the end of all local control measures until a respective event occurs or last patient contact, at which time the patient was censored. Patients who experienced disease progression, second malignant neoplasm, or death were scored as having experienced an event.

### Statistics

The outcome measures were OS, EFS, DFS, and cumulative incidence (CI) of LF and DF timed from the completion of local control therapy, as calculated by the Kaplan-Meier method. The CI of each type of event was calculated for each method of local control and compared by the log-rank test. Associations between categorical variables were analyzed using Pearson’s chi-square test. The Mann-Whitney test was used to compare medians for radiation dose. The association between local control modality and event risk was analyzed using univariate and multivariate Cox proportional-hazards regression models. The hazard ratio (HR) and 95% confidence interval (95% CI) were used as the measure of effect.

## Results

### Patients selection and characteristics

Data from 73 patients (45 males and 28 females, mean age of 12.8 years) with localized bone disease submitted to local control were selected from a total of 175 patients (96 with localized bone and extraosseous ES and 79 with metastatic bone and extraosseous ES) of the EWING1 trial. The median follow-up time of patients in this study was 4.5 years (range, 2.3 to 6.7 years). Forty-three tumors (58.9%) were located in the extremities, 10 (13.7%) in the pelvis, 10 (13.7%) in the chest wall, 6 (8.2%) in the spine, and 4 (5.5%) in other sites (*p* > 0.001). Thirty-eight (52.1%) patients were allocated as LRG and 35 (47.9%) patients as HRG (*p* < 0.001). Pelvic tumors were relatively more likely to receive radiotherapy than surgery alone. On the other hand, non-pelvic tumors were more frequently treated with surgery *(p* = 0.012*).* Tumor size ≤ 8 cm vs > 8 cm was not significantly associated with the local control modality performed (*p =* 0.12). The response to chemotherapy was poor (necrosis index ≤ 95%) in 56% and good (> 95%) in 44% of patients. Of 68 patients with complete LDH records, only 15 (22%) had LDH ≥ 1.5 x ULN and were more likely to have a surgical procedure (66.6%) than radiotherapy alone (33.3%) (*p =* 0.05). The median radiation dose was 50.4 Gy for both groups (range, 45.0 to 55.9 Gy).

Of 43 patients with tumors of the extremities, almost all underwent surgical treatment (*n* = 41, 95.4%), while only 2 (4.6%) received radiotherapy alone. Of 16 patients with tumors of the pelvis and spine, only 6 (37.5%) underwent surgery, while 10 (62.5%) received radiotherapy alone (*p* < 0.001) (Table 1).

**Table 1:** Characteristics of the Sample according to the Local Control Modality

Variables	Total sample	Local Control Modality			*p*
		Surgery	Surgery + Radiotherapy	Radiotherapy	
	*n* (%)	*n* (%)	n (%)	n (%)	
All patients	73 (100)	47 (64.4)	13 (17.8)	13 (17.8)	
Age group					0.753
≤ 15 years	51 (69.9)	34 (72.3)	8 (61.5)	9 (69.2)	
> 15 years	22 (30.1)	13 (27.7)	5 (38.5)	4 (30.8)	
Sex					0.035
Male	45 (61.6)	33 (70.2)^a^	4 (30.8)	8 (61.5)	
Female	28 (38.4)	14 (29.8)	9 (69.2)^a^	5 (38.5)	
Risk group					<0.001
Low	38 (52.1)	33 (70.2)^a^	5 (38.5)	0 (0.0)	
High	35 (47.9)	14 (29.8)	8 (61.5)	13 (100)^a^	
Tumor size					0.124
≤ 8 cm	18/45 (40.0)	10/31 (32.3)	4/9 (44.4)	4/5 (80.0)	
> 8 cm	27/45 (60.0)	21/31 (67.7)	5/9 (55.6)	1/5 (20.0)	
Necrosis Index					1.000
≤ 95%	28/50 (56.0)	23/42 (54.8)	5/8 (62.5)	NA	
> 95%	22/50 (44.0)	19/42 (45.2)	3/8 (37.5)	NA	
LDH					0.052
≥ 1.5 x ULN	15/68 (22.1)	6/45 (13.3)	4/10 (40.0)	5/13 (38.5)	
< 1.5 x ULN	53/68 (77.9)	39/45 (86.7)	6/10 (60.0)	8/13 (61.5)	
Recurrence					0.509
No	53/68 (77.9)	34/46 (73.9)	10/12 (83.3)	9/10 (90.0)	
Local	4/68 (5.9)	3/46 (6.5)	0/12 (0.0)	1/10 (10.0)	
Systemic	11/68 (16.2)	9/46 (19.6)	2/12 (16.7)	0/10 (0.0)	
Site location					<0.001
Spine	6 (8.2)	0 (0.0)	1 (7.7)	5 (38.5)^a^	
Chest wall	10 (13.7)	6 (12.8)	4 (30.8)^a^	0 (0.0)	
Pelvis	10 (13.7)	3 (6.4)	2 (15.4)	5 (38.5)^a^	
Proximal extremity	21 (28.8)	17 (36.2)^a^	2 (15.4)	2 (15.4)	
Distal extremity	22 (30.1)	19 (40.4)^a^	3 (23.1)	0 (0.0)	
Other	4 (5.5)	2 (4.3)	1 (7.7)	1 (7.7)	
Radiation dose (Gy)^b^	50.4 (45–55.8)	NA	50.4 (45–54.9)	50.4 (45–55.8)	0.801

^a^Statistically significant association by adjusted residual analysis at 5% significance level

^b^Expressed as median (25th–75th percentile)

*LDH* lactate dehydrogenase, *NA* not applicable, *ULN* upper limit of normal

### Overall analysis

**Fig. 1:**
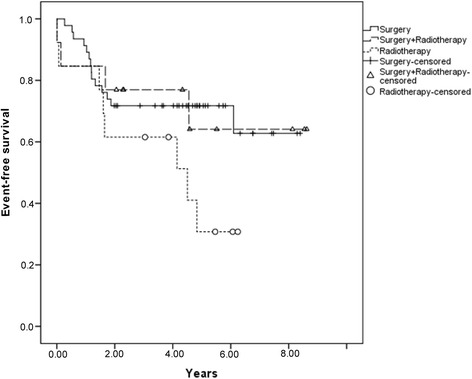
Event-free survival according to the local treatment modality

**Fig. 2:**
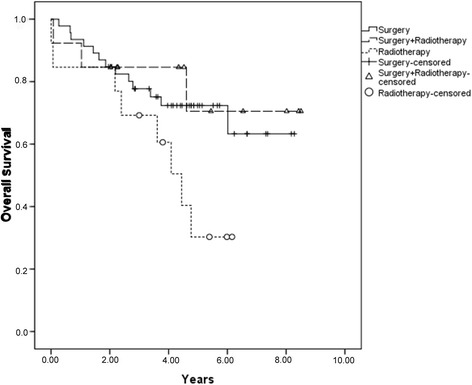
Overall survival according to the local treatment modality

**Fig. 3:**
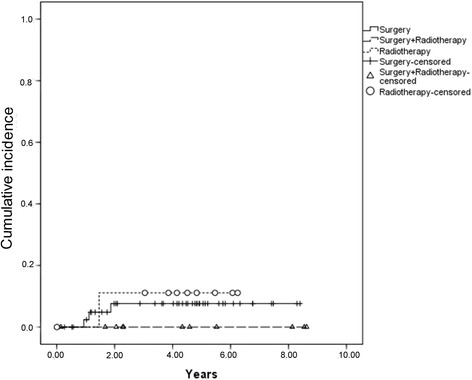
Cumulative incidence of isolated local recurrence in relation to local treatment modality

The estimated 5-year EFS, OS, and DFS for all 73 patients was 62.1%, 63.3%, and 73.1%, respectively*.* The 5-year CI of LF and DF was 6.9% and 14.7%, respectively. Sixty-eight patients had complete information on local or distant recurrence. Only 4 had isolated LF, and 11 had DF combined or not with LF (Table 2; Figs. 1, 2, and 3).

**Table 2:** Results of univariate analysis for possible independent variables associated with death and EFS

Variables	EFS			LF		
	5-year CI	HR (95% CI)	*p*	5-year CI	HR (95% CI)	*p*
All patients	62.1%	-	-	6.9%	-	-
Age group						
≤ 15 years	68.0%	1.00		4.6%	1.00	
> 15 years	47.6%	2.00 (0.91–4.41)	0.087	13.8%	3.11 (0.44–22.1)	0.257
Sex						
Male	64.0%	1.00		9.1%	1.00	
Female	58.6%	1.30 (0.60–2.83)	0.513	4.8%	0.58 (0.06–5.53)	0.575
Risk group						
Low	73.7%	1.00		5.6%	1.00	
High	48.2%	1.74 (0.80–3.80)	0.163	8.3%	1.33 (0.19–9.41)	0.779
Tumor size						
≤ 8 cm	61.1%	1.00		--	--	--
> 8 cm	58.1%	1.07 (0.41–2.76)	0.892	--	--	--
Necrosis Index						
≤ 95%	60.7%	1.38 (0.54–3.57)	0.503	5.6%	0.41 (0.04–4.52)	0.466
> 95%	71.3%	1.00		10.5%	1.00	
LDH						
≥ 1.5 x ULN	51.3%	1.11 (0.44–2.77)	0.832	15.4%	6.04 (0.55–66.7)	0.142
< 1.5 x ULN	63.1%	1.00		2.4%	1.00	
Pelvic location						
Yes	41.1%	1.47 (0.55–3.90)	0.440	--	--	--
No	66.7%	1.00		--	--	--
Radiation dose	-	0.99 (0.98–1.01)	0.560	-	0.98 (0.86–1.12)	0.796

*EFS* event-free survival, *LF* local failure, *CI* cumulative incidence, *HR* hazard ratio; 95% CI, 95% confidence interval, *LDH* lactate dehydrogenase, *ULN* upper limit of normal

The 5-year EFS was not statistically different according to tumor size ≤ 8 cm vs > 8 cm at presentation (61.1% vs 58.1%, HR = 1.07; *P =* 0.89), pelvic location (41.1% vs 66.7%, HR = 1.47; *p =* 0.44), LDH levels < 1.5 vs ≥ 1.5 x ULN (63.1% vs 51.3%, HR = 1.11; *p* = 0.83), or radiation dose (HR = 0.99; *p =* 0.56). LRG and HRG patients had EFS rates of 73.7% and 48.2% and LF rates of 5.6% and 8.3%, respectively (*p* = 0.16) (Table 2).

On multivariate analysis, definitive radiotherapy, age > 15 years and HRG were not associated with a higher risk of any event (Table 3).

**Table 3:** Results of multivariate analysis for independent variables associated with death and EFS

	EFS	*p*	LF	*p*
Variables	HR (95% CI)		HR (95% CI)	
Type of treatment				
Surgery	1.00		1.00	
Surgery + Radiotherapy	0.88 (0.28–2.74)	0.829	*	*
Radiotherapy	1.84 (0.63–5.41)	0.267	1.01 (0.08–12.8)	0.991
Age group				-
≤ 15 years	1.00		-	
> 15 years	2.12 (0.96–4.71)	0.064	-	
Risk group				-
Low	1.00		-	
High	1.41 (0.53–3.71)	0.489	-	
LDH				
≥ 1.5 x ULN	-		6.28 (0.50–79.1)	0.155
< 1.5 x ULN	-		1.00	

*It is not possible to estimate risk because the interval tends to infinity

*EFS* event-free survival, *LF* local failure, *HR* hazard ratio, 95% CI, 95% confidence interval, *LDH* lactate dehydrogenase, *ULN* upper limit of normal

### Local control analysis

The 5-year EFS was 30.8% for patients submitted to definitive radiotherapy (13 patients), 64.1% for surgery plus radiotherapy (13 patients), and 71.7% for surgery alone (47 patients) (*p =* 0.009)*.* There was no significant difference in LF rates by local control modality (*p* = 0.61), and the LF rates were the same at 2 and 5 years of follow-up: 7.6% in the surgery group, 11% in the radiotherapy group, and 0% in the PORT group (*p* = 0.62). Considering all 15 patients with local or systemic recurrence, the CI of LF and DF at both 2 and 5 years was 11% for radiotherapy alone, 16.7% for surgery plus radiotherapy, and 25% for surgery alone (*p* = 0.64). The local disease control rate was 78%.

## Discussion

Small round cells tumors such as ES are usually good responders to irradiation. Consequently, radiotherapy has been an important option for local control either alone or with surgery. However, radiotherapy is not free from complications at the primary tumor sites. Soft tissue fibrosis, osteonecrosis, impaired long-bone growth, secondary malignancies, and up to 35% rate of local recurrence have been related to high-dose irradiation [5, 9–13, 23].

On the other hand, development of orthopedic endoprostheses has enabled surgeons to perform non-mutilating procedures with adequate margins in ES patients. Continuous advances have introduced structural auto and allografts in surgical reconstructions, thus offering more biological treatment options. Therefore, amputation has become extremely infrequent in ES [24, 25].

The presence of marginal or contaminated margins is still the main indication for PORT in the treatment of ES. Conversely, PORT has been routinely used in patients with poor response to chemotherapy as well as in large-volume tumors in European centers. The current consensus on the type of local treatment of ES follows criteria based on the patient and tumor characteristics and, not less important, on the level of experience of treating physicians [12–19].

The heterogeneity of clinical factors may be a source of confusion when following the guidelines for local treatment in ES [6, 26]. Yock et al. evaluated the impact of the local control modality for localized ES in a non-randomized study including 75 patients with pelvic bone disease. There was no difference in recurrence rates or survival between the different local control methods. However, patients with larger tumors were more likely to receive combined surgery plus radiotherapy (*p* = 0.013) [19]. Similarly, in the EWING1 trial, there was no difference in recurrence rate (LF) between the different treatment modalities, and larger tumors were more likely to receive surgery and PORT than radiotherapy (*p* = 0.12). Nevertheless, we believe that the limited size of the sample and the inability to control for confounding factors may be reflected in the results.

Surgery is reserved for situations in which the tumor can be resected with adequate margins, that is, with no evidence of residual disease. Although based on observational studies, local recurrence and survival have shown better results in patients submitted to neoadjuvant chemotherapy and surgery compared to patients submitted to neoadjuvant chemotherapy and radiotherapy [16, 27, 28]. DuBois et al. analyzed using propensity scores the risk of LF and survival in 465 patients with localized ES of bone and found that radiotherapy had a higher risk of local recurrence and death than surgery alone [13]. In the EWING1 trial, radiation therapy showed worse results in terms of EFS (*p* = 0.009) than surgery and PORT. These findings should be analyzed with caution because 70% (9/13) of the patients subjected to radiation had unresectable tumors; 10 patients had tumors located in the spine and pelvis and 3 developed secondary myeloproliferative neoplasms at the beginning of the follow-up period. Due to the small number of local recurrences (*n* = 4), there was no significant difference in LF rates by local control modality.

Several studies included only patients with pelvic ES to investigate possible associations between local control modality and treatment failure [19, 29–31]. Raciborska et al. found that survival was higher in patients treated with surgery and PORT than in those treated with radiotherapy alone (81% and 78% vs 36% at 3 years, respectively) [29]. In the present study, 10 patients had pelvic tumors, and 50% of these patients were treated with definitive radiotherapy (*p* = 0.012). As expected, survival was considerably lower in patients with pelvic compared to non-pelvic tumors (41.1% vs 66.7%, *p* = 0.44). There was no difference in the incidence of LF and survival between the different local control measures in the pelvis.

Nowadays, definitive radiation is an almost exclusive indication for unresectable tumors and for patients with poorer prognosis for whom surgical procedures may be exceptionally mutilating. Advances in radiation technology and multidisciplinary approach have enhanced local control and decreased complications in healthy tissues surrounding tumors. Studies analyzing the use of radiation alone reported 5-year local control rates ranging from 53 to 86% with doses between 45 and 65 Gy [9–11, 26, 32, 33]. The EWING1 trial demonstrated that most patients with unresectable tumors and tumors located in the spine and pelvis were treated with definitive radiotherapy. Considerably worse results were obtained in patients treated with radiotherapy alone than in those treated with surgery and PORT. This may be due to high disease morbidity, suboptimal local control with radiotherapy alone, or a combination of these and other factors. The differing clinical characteristics of the radiotherapy group precluded a perfectly reliable comparison between the different local treatment modalities.

Moreover, EWING1’s sample was characterized by patients with many risk factors associated with poor prognosis. Forty-eight percent were in the HRG, and more than half had tumors >8 cm and were poorer responders to chemotherapy. These worse characteristics suggest a delay in ES diagnosis probably related to social and economic issues from a developing country. Furthermore, higher resistance to chemotherapy could be related to both larger tumors and a specific resistance profile of the patients. Despite all this, for 73 patients included in the current study, the remission rate was 78%.

In summary, we observed similar results to those published by large international cooperative groups [5, 16, 19, 34]. Every effort made to provide training to local investigators, gather data, and monitor the progress of the first Brazilian protocol for ES has allowed us to describe the different local control strategies used in the treatment of ES in a country of continental size like Brazil. The great economic, cultural and social diversity of patients as well as the different levels of knowledge of health professionals on the topic make clear the importance of a collaborative approach for a study of this magnitude.

## Conclusion

The EWING1 trial found no significant difference in local or systemic disease recurrence between different treatment modalities. However, regarding survival, there was a significant difference between surgery, radiotherapy, and PORT.

The Brazilian Collaborative Study Group for treatment of ESFT has now been incorporated into the newly formed Latin American Pediatric Oncology Group (GALOP) and a second ESFT study was activated in 2011 [28]. The next step is intended to analyze and report the impact of local control in the second ESFT study.

## Abbreviations

95% CI: 95% confidence interval
CI: Cumulative incidence
CT: Computed tomography
DF: Distant failure
EFS: Event-free survival
ES: Ewing sarcoma
ESFT: Ewing sarcoma family of tumors
HR: Hazard ratio
HRG: High-risk group
ICE: Ifosfamide, carboplatin, and etoposide
IE: Ifosfamide and etoposide
LDH: Lactate dehydrogenase
LF: Local failure
LRG: Low-risk group
MRI: Magnetic resonance imaging
OS: Overall survival
ULN: Upper limit of normal
VDC: Vincristine, doxorubicin, and cyclophosphamide
